# Targeted Therapies for EGFR Exon 20 Insertion Mutation in Non-Small-Cell Lung Cancer

**DOI:** 10.3390/ijms25115917

**Published:** 2024-05-29

**Authors:** Donghyun Seo, Jun Hyeok Lim

**Affiliations:** 1 Department of Medicine, Inha University College of Medicine, Incheon 22332, Republic of Korea; 2 Division of Pulmonology, Department of Internal Medicine, Inha University Hospital, Inha University College of Medicine, Incheon 22332, Republic of Korea

**Keywords:** non-small-cell lung cancer, EGFR exon 20 insertion, tyrosine kinase inhibitors, amivantamab, mobocertinib, poziotinib, zipalertinib, sunvozertinib

## Abstract

Non-small-cell lung cancer (NSCLC) frequently harbors mutations in the epidermal growth factor receptor (EGFR), with exon 20 insertions comprising 1–10% of these mutations. EGFR exon 20 insertions are less responsive to conventional tyrosine kinase inhibitors (TKIs), leading to the development of targeted agents. This review explores key therapeutic agents, such as Amivantamab, Mobocertinib, Poziotinib, Zipalertinib, and Sunvozertinib, which have shown promise in treating NSCLC with EGFR exon 20 insertions. Amivantamab, a bispecific antibody-targeting EGFR and c-MET, demonstrates significant efficacy, particularly when combined with chemotherapy. Mobocertinib, a TKI, selectively targets EGFR exon 20 mutations but faces limitations in efficacy. Poziotinib, another oral TKI, shows mixed results due to mutation-specific responses. Zipalertinib and Sunvozertinib have emerged as potent TKIs with promising clinical data. Despite these advances, challenges in overcoming resistance mutations and improving central nervous system penetration remain. Future research should focus on optimizing first-line combination therapies and enhancing diagnostic strategies for comprehensive mutation profiling.

## 1. Introduction

In non-small-cell lung cancer (NSCLC), mutations in the epidermal growth factor receptor (EGFR) are notably prevalent, occurring in approximately 30% of cases [1]. Among these, the L858R point mutation in exon 21 and deletions involving at least three amino acids in exon 19 are classified as classical EGFR activating mutations, constituting 85–90% of all mutations within the EGFR kinase domain observed in NSCLC [2]. In contrast, insertions in exon 20 of EGFR, which account for roughly 1–10% of all EGFR mutations, are associated with a reduced efficacy of tyrosine kinase inhibitors (TKIs) and represent the third most common type of EGFR mutation in NSCLC. These mutations are more frequently identified in patients who have never smoked, are female, and present with adenocarcinoma histology [3,4,5,6].

Insertions in EGFR exon 20 are primarily located immediately posterior to the αC-helix within the tyrosine kinase domain, with these mutations comprising between 1% and 9% of all EGFR mutations [4,6,7,8,9]. The region of exon 20 spans amino acids 762 to 775, which includes the αC-helix from amino acids 762 to 766 and the subsequent loop up to amino acid 775 [4,10,11,12,13,14]. Predominantly, these insertion mutations (90%) impact the loop area following the αC-helix, ranging from amino acids 767 to 775. Within this spectrum, mutations found in the loop closer to the αC-helix and those in the far loop form two distinct molecular subgroups [15]. A smaller portion (10%) of these mutations affects the portion of the αC-helix closer to the C-terminal [4,12]. The mutations in exon 20 are diverse, typically manifesting as in-frame insertions or duplications that can range from 3 to 21 base pairs, corresponding to 1 to 7 amino acids [4,12]. These structural changes cause the αC-helix and the P-loop to shift towards the drug-binding pocket, which creates significant spatial interference, thereby reducing the effectiveness of traditional EGFR tyrosine kinase inhibitors [16].

The αC-helix within the EGFR tyrosine kinase domain plays a pivotal role in regulating the receptor’s activity, transitioning between outward and inward orientations to enable interactions that promote the stabilization of its dimerization-ready state [17]. In the context of exon 19 deletions, these mutations lead to the loss of specific amino acid residues from the loop preceding the αC-helix on the N-terminal side, effectively shortening the loop. This modification is thought to constrain the αC-helix’s mobility, skewing it towards an inward, active configuration and thus facilitating continuous receptor activation. Conversely, insertions in exon 20, located at the C-terminal end of the αC-helix or more frequently within the ensuing loop, are believed to exert an opposite effect. These insertions are suggested to drive the αC-helix into a similarly active state, but by pushing from the opposite direction, thereby supporting the hypothesis of induced active conformations through structural manipulation of the α-helix.

Research indicates that mutations in EGFR exon 20 often do not cooccur with other EGFR mutations, suggesting a distinct mutational pathway in NSCLC [6,7,8,9,18]. The heterogeneity of these exon 20 insertion mutations significantly affects the molecular interaction dynamics within the EGFR, altering both drug and ATP binding, which, in turn, influences the efficacy of EGFR-targeted therapies. Typically, these mutations confer resistance to first- and second-generation TKIs, such as erlotinib and gefitinib [3,6,8,9,12,19,20]. An exception is found in the A763_Y764insFQEA mutation, which differs structurally from other exon 20 insertions. This particular mutation alters the β3-αC loop, enhancing the loop’s extension and thus increasing the catalytic efficiency of the mutated receptor, akin to the activation seen with the L858R mutation in exon 21. This alteration underpins the atypical sensitivity of the A763_Y764insFQEA mutation to EGFR TKIs, contrasting with the typical resistance observed with other exon 20 insertions.

This review article explores diagnostic technologies for lung cancer with EGFR exon 20 insertion mutations and summarizes the latest clinical studies on therapeutic developments. It goes a step further than previous review articles by incorporating the most recent updates on research trends, ongoing clinical trials, and future directions in the treatment of this cancer variant.

## 2. Detection Methods for EGFR Exon 20 Insertion (Table 1)

Polymerase chain reaction (PCR) and next-generation sequencing (NGS) are two primary methodologies employed for the detection of EGFR mutations [21]. PCR assays, renowned for their rapid turnaround and sensitivity, are broadly accessible [22]. However, due to the high variability in EGFR exon 20 insertion mutations, conventional PCR techniques often fail to detect more than 50% of these mutations, underscoring the critical role of NGS in identifying such genetic anomalies [23]. Typically, PCR kits are calibrated only for the most common EGFR mutations, contributing to the notable under-detection of exon 20 insertion variants. Comparative analyses between allele-specific PCR and NGS demonstrate that EGFR exon 20 insertion mutations are frequently missed, thereby highlighting the superior capability of NGS in delineating the extensive spectrum of EGFR exon 20 insertion in NSCLC [24]. This methodological preference is further justified by NGS’s ability to identify the specific types of EGFR exon 20 insertions, which is vital due to the considerable heterogeneity of these mutations and the variable efficacy of certain exon 20 insertion-specific EGFR TKIs and earlier generation TKIs. Liquid biopsy, specifically, the analysis of cell-free DNA via next-generation sequencing, has the potential to enhance detection rates for EGFR exon 20 insertions and other rare oncogenic mutations in advanced NSCLC, a disease often associated with limited tissue availability. Tissue and plasma should be recognized as complementary tools for tumor genotyping. Leveraging all available resources can broaden the identification of clinically relevant biomarkers associated with approved targeted therapies, thereby ensuring that every eligible patient receives potential benefits from these treatments. NGS-based companion diagnostics that analyze circulating tumor DNA from plasma, such as Guardant360 and FoundationOne Liquid CDx, offer the potential to obviate the need for biopsy and facilitate mutation detection in cases with limited available tumor tissue [25]. Guardant360 CDx, an NGS-based diagnostic tool, uses specialized high-throughput hybridization-based capture technology to detect specific EGFR exon 20 insertions, known as ex20ins, from circulating tumor DNA in blood plasma. These insertions occur between codons 763 and 773 of the EGFR gene. Similarly, FoundationOne Liquid CDx, alongside Guardant360 CDx, employs a hybrid-capture approach with probes within an NGS library, ensuring high accuracy and sequencing depth. These assays are adept at identifying a range of genetic changes pertinent to NSCLC, including point mutations, small insertions and deletions, crucial fusions, and copy number variations. In contrast to PCR-based techniques, plasma NGS offers the unique ability to detect both well-documented mutations as well as rare or novel variants.

**Table 1:** Comparison of PCR and NGS for detection of EGFR exon 20 insertion.

Methods	Advantages	Limitations
PCR	- Widely available in most medical facilities - Low costs and turnaround time (TAT) - Suitable for single or a few mutation tests	- Risks missing a significant proportion of EGFR exon 20 insertions - Incomplete tumor genotyping in most of the patients - High TAT for all validated biomarkers
NGS	- Low TAT for all the validated biomarkers - Cost-effective for a complete tumor genotyping - Higher coverage for rare and new variants - Capable of analyzing multiple genomic alterations - Identification of co-mutations - High sensitivity and specificity, contributing to precision medicine	- Higher costs and TAT - Requires advanced technical skills for data analysis and storage

The exon 20 insertion mutation in EGFR adds amino acids within the kinase domain, altering its structure. This structural change can permanently shift the active site to an “on” state. Essentially, the mutation mimics continuous recognition of growth factors by EGFR, maintaining it in a perpetually active state. The altered structure of the kinase domain leads to the continuous activation of signaling pathways mediated by EGFR. EGFR predominantly activates the RAS/RAF/MEK/ERK and PI3K/AKT signaling pathways, which promote cellular survival, growth, and proliferation. The sustained activation of EGFR contributes to increased cell division and growth in cancer cells, as well as increased resistance to apoptosis. Consequently, this plays a role in cancer progression and metastasis. Studies have shown that the exon 20 insertion mutation exhibits a more potent oncogenic effect compared to other EGFR mutations [4,12]. For example, in tumor cell models, EGFR with this mutation has been shown to increase cellular growth and enhance resistance to anticancer drugs. This continuous activation reduces the effectiveness of standard EGFR inhibitors. Most EGFR inhibitors are designed to bind to the inactive state of EGFR, thus they fail to effectively inhibit the constantly active mutant EGFR.

## 3. Targeted Agents against EGFR Exon 20 Insertion (Table 2)

### 3.1. Amivantamab

Amivantamab, a fully human bispecific monoclonal antibody targeting both EGFR and c-MET, exhibits significant antitumor activity through its dual targeting design [26]. It suppresses tumor growth by interfering with ligand–receptor interactions and blocking ligand-induced phosphorylation of EGFR and c-MET. Additionally, amivantamab enhances immune-mediated tumor cell destruction by inducing Fc-mediated phagocytosis and natural killer cell cytotoxicity against cells expressing EGFR and c-MET [27,28,29].

**Table 2:** Results of the clinical trials for patients with EGFR exon 20 insertion.

Agent	Phase	N	Patient Population	Primary Endpoint	ORR	DCR	DOR, Months	PFS, Months	OS, Months
Poziotinib [30]	II	115	advanced NSCLC with an EGFR exon 20 insertion who had received at least one prior line of therapy	ORR	14.8%	68.7%	7.4	4.2	NA
Poziotinib [31]	II	79	treatment-naïve metastatic NSCLC patients with EGFR exon 20 mutations	ORR	27.8%	86.1%	9.2	7.2	NA
Mobocertinib [32]	I/II	28	previously treated locally advanced or metastatic NSCLC	ORR	43%	86%	13.9	7.3	NA
Mobocertinib [33]	I/II	96	EGFR ex20ins mutation, and 1 or 2 prior regimens of systemic anticancer chemotherapy for locally advanced or metastatic disease	ORR	25%	78%	17.5	7.3	24
Mobocertinib [34]	III	354	untreated EGFR ex20ins+ locally advanced/metastatic NSCLC	PFS	32%	87%	NA	9.59	NA
Amivantamab [29]	I	81	patients who harbored EGFR exon20ins mutations, after platinum-based chemotherapy	Dose-limiting toxicity, ORR	40%	NA	11.1	8.3	22.8
Amivantamab + chemotherapy [35]	III	308	patients had received no previous treatment for locally advanced or metastatic NSCLC with EGFR exon 20 insertion	PFS	73%	92%	9.7	11.4	NA
Zipalertinib [36]	I/IIa	73	patients with advanced, EGFR ins20-mutant NSCLC received prior platinum-based chemotherapy	ORR, DOR	36%	85%	>15	12	NA
Sunvozertinib [37]	I/II	52	locally advanced or metastatic NSCLC patients harboring EGFR Exon20ins post platinum treatment	ORR	50%; 55.6%; 44.8%; 22.2%	NA	NA (200 mg); 5.6 (300 mg)	PFS rate: 50%; 53.3%; 44.6%; 44.4%	NA

Drawing on preclinical data, the CHRYSALIS trial was a phase I clinical study involving previously treated NSCLC patients with exon 20 insertion mutations [29]. Amivantamab significantly reduces the viability of cell lines (Ba/F3) and patient-derived organoids harboring EGFR exon 20 insertions [38]. The CHRYSALIS trial indicated a median overall survival of 22.8 months, and a median progression-free survival of 8.3 months, and an objective response rate of 40%. The frequently observed adverse events were rash (86%), infusion reactions (66%), and paronychia (45%). Given that the study cohort comprised individuals with relapsed, metastatic, or inoperable NSCLC and a 5-year survival rate under 10%, these results are of substantial clinical significance. Consequently, the FDA has approved amivantamab for the treatment of NSCLC patients with EGFR exon 20 insertion mutations who have progressed following platinum-based chemotherapy.

The mechanism of amivantamab potentially explains its enhanced efficacy and reduced toxicity, when compared to other targeted therapies for NSCLC harboring EGFR exon 20 insertions [39]. Amivantamab is administered intravenously, starting with weekly doses before moving to a biweekly schedule, while mobocertinib is administered orally on a daily basis. While the response rate for amivantamab was higher than that for mobocertinib, caution is warranted in making direct comparisons across trials. Notably, the overall survival and progression-free survival outcomes were comparable for amivantamab and mobocertinib. Infusion reactions of all grades were observed in 65% of patients treated with amivantamab, and 3% experienced grade 3 or higher reactions (Grade 1, mild reactions such as slight rash, flushing, or nasal congestion, usually requiring no treatment; Grade 2, moderate symptoms like transient rash or urticaria, moderate flushing, or chest/back pain, typically necessitating symptomatic treatment; Grade 3, severe reactions involving symptoms such as severe rash with associated symptoms, or significant hypotension, often leading to drug interruption; Grade 4, life-threatening symptoms like anaphylaxis requiring urgent intervention). Amivantamab is more commonly associated with skin rash and less frequently induces diarrhea compared to mobocertinib.

The promising outcomes from the phase I CHRYSALIS study have led to the phase III PAPILLON study, a randomized open-label trial evaluating the efficacy of amivantamab combined with chemotherapy versus chemotherapy alone as a first-line treatment for EGFR exon 20 insertion mutations in NSCLC patients [35]. This trial randomized 308 patients to receive carboplatin and pemetrexed, with or without amivantamab, until disease progression. The ORR was notably higher for the combination arm (73% vs. 43%) with more durable responses (median DoR of 9.7 months vs. 4.4 months). The PFS was significantly longer in the combination arm (median PFS of 11.4 months vs. 6.7 months). The combination therapy demonstrated superior efficacy, with an 18-month PFS of 31% versus 3% for chemotherapy alone. Median OS displayed a favorable trend for the combination arm, with a survival rate of 24.4 months in the chemotherapy group and no median reached in the amivantamab group. Safety profiles were similar across both arms, with serious adverse events occurring in 37% and 31% of patients in the amivantamab and chemotherapy monotherapy arms, respectively. The frequent adverse events were infusion-related reactions (42%), cutaneous toxicity (paronychia 56% and rash 54%), peripheral edema (30%), and hypoalbuminemia (41%). Dose reductions occurred in 36% of amivantamab recipients, while discontinuation due to adverse events was similar in both arms.

### 3.2. Mobocertinib

Mobocertinib is an oral TKI developed to specifically target EGFR exon 20 insertion mutations. An unoccupied pocket was identified through a docking model that persists following the binding of the EGFR ex20insNPG mutant to osimertinib, which can be accessed via substitutions at the pyrimidine ring. Hence, mobocertinib’s isopropyl ester was uniquely designed to engage with the gatekeeper residues within this pocket. Additionally, mobocertinib establishes an irreversible bond with the Cys797 residue in EGFR, forming a covalent interaction, which enhances its binding affinity [40,41,42].

Preclinical in vitro studies revealed that mobocertinib selectively inhibits mutant EGFR over wild-type EGFR, demonstrating more potent inhibition of Ba/F3 cells expressing EGFR exon 20 insertions than wild-type EGFR. Additionally, it exhibited in vivo antitumor efficacy in patient-derived and murine orthotopic models [41,43]. This led to the evaluation of mobocertinib in a phase I/II dose-escalation and expansion study, which established 160 mg/day as the recommended phase II dose [32]. The study produced encouraging results, with an ORR of 28%, a median DOR of 17.5 months, a median PFS of 7.3 months, and a median OS of 24.0 months in the platinum-pretreated cohort [33,34]. In September 2021, based on data from this phase I/II study, the FDA approved mobocertinib as the first oral therapy specifically designed for patients with NSCLC harboring EGFR exon 20 insertion mutations.

The frequent adverse events were skin rash, paronychia, decreased appetite, diarrhea, nausea, vomiting, and stomatitis, occurring in over 20% of patients. Most gastrointestinal and dermatologic events were grade 1–2 in severity, with diarrhea being the only treatment-related grade 3–4 AE reported in more than 10% of patients. A treatment-related death due to heart failure was reported, and the drug’s labeling includes a boxed warning for QTc prolongation and Torsades de Pointes [33].

The phase III EXCLAIM-2 trial assessed the efficacy of first-line mobocertinib compared to platinum-based chemotherapy in patients with NSCLC harboring EGFR exon 20 insertions and who had not received prior therapy [34]. The results showed that mobocertinib had comparable efficacy to first-line platinum-based chemotherapy but did not demonstrate superiority. Consequently, due to its failure to meet the primary endpoint of PFS, mobocertinib was withdrawn as a treatment option for NSCLC patients with EGFR exon 20 insertion mutations.

### 3.3. Poziotinib

Poziotinib is an oral quinazoline-based inhibitor of EGFR, functioning as an irreversible pan-human epidermal growth factor receptor (HER) inhibitor with activity against mutations or insertions in HER1, HER2, and HER4. Its flexibility, small molecular size, and enhanced halogenation enable poziotinib to overcome steric hindrances within the drug-binding pocket of EGFR exon 20 insertion mutations. In xenograft models derived from patients with EGFR or HER2 exon 20 mutant NSCLC and in genetically engineered NSCLC mouse models, poziotinib demonstrated superior activity compared to currently approved EGFR TKIs [16].

Although poziotinib demonstrates efficacy against EGFR exon 20 insertion mutations, response heterogeneity may arise depending on the mutation’s location within EGFR exon 20. This variability is attributed to significant conformational differences in specific receptor regions that impact drug binding. Mutations located near the loop region adjacent to the C-helix influence the positioning of particular residues in the P-loop, thereby stabilizing poziotinib and enhancing its binding affinity. This behavior differs from insertions situated in the distal far loop region. Poziotinib’s response rate is 46% for near-loop mutations, compared to 0% for far-loop mutations. In vitro testing has similarly shown a strong correlation between mutation location and drug sensitivity [44].

The ZENITH20 trial, a multicenter phase II study, assessed the efficacy and safety of poziotinib in NSCLC patients with EGFR or HER2 exon 20 insertion mutations. Cohort 1 included 115 NSCLC patients with EGFR exon 20 insertions who had previously received treatment with poziotinib [30]. The study reported an ORR of 14.8% and a DCR of 69%, with a median PFS of 4.2 months and a median DOR of 7.4 months. Although the primary endpoint of ORR was not met, poziotinib did shrink tumors in 65% of patients. Cohort 3, consisting of 79 patients with EGFR exon 20 insertions treated with first-line poziotinib, showed an ORR of 27.8%, DCR of 86.1%, median PFS of 7.2 months, and median DOR of 9.2 months [31]. Despite the clinically meaningful activity reported in the ZENITH20 study for previously treated NSCLC with EGFR exon 20 insertion mutation [45], significant adverse events were observed due to inhibition of wild-type EGFR. Notably, 28% and 26% of patients experienced grade 3 or higher rash and diarrhea, respectively. Treatment-related pneumonitis occurred in 4% of patients, although some cases could have been influenced by prior treatment with checkpoint inhibitors. In the ZENITH20-5 study, patients were randomized to receive either 10, 12, or 16 mg once daily, or 6 or 8 mg twice daily of poziotinib. Comparing randomized cohorts (16 mg QD vs. 8 mg BID; 12 mg QD vs. 6 mg BID), BID dosing in the first cycle resulted in a lower frequency of grade 3 or higher adverse events, including rash, diarrhea, and stomatitis [31,46].

### 3.4. Zipalertinib

Zipalertinib, a newly developed oral, irreversible EGFR TKI, is characterized by its distinct pyrrolopyrimidine structure and exhibits potent, broad-spectrum efficacy against EGFR mutations [47,48]. Its distinguishing feature lies in its pronounced potency and specificity in inhibiting EGFR exon 20 insertion mutations compared to wild-type EGFR. Like mobocertinib, Zipalertinib irreversibly binds to the Cys-797 residue of EGFR proteins carrying ex20ins mutations. In vitro kinase assays have confirmed that Zipalertinib selectively targets D770_N771insNPG while sparing wild-type EGFR, suggesting an enhanced therapeutic window [47]. Furthermore, preclinical research has shown that Zipalertinib specifically inhibits Ba/F3-engineered cell lines harboring various EGFR ex20ins mutations [48].

In light of the preclinical findings, the clinical efficacy of Zipalertinib was assessed in a phase I/IIa trial (NCT04036682). The preliminary results from 25 patients with NSCLC containing EGFR exon 20 insertion mutations revealed that 10 patients (40%) exhibited a partial response (PR), 14 (56%) had stable disease (SD), and 1 (4%) had progressive disease (PD) as their best response. Additionally, 82% of patients experienced tumor reduction [49]. The most frequently observed treatment-related adverse events of all grades included rash (49%), diarrhea (24%), and paronychia (16%), while grade 3 adverse events comprised anemia (5%) and diarrhea (3%).

The phase I/II dose-escalation and dose-expansion trial’s preliminary results included 73 patients at dose levels ranging from 30 to 150 mg BID [36]. Enrollment for the 150 mg BID dose was halted after 11 patients due to toxicity concerns. Among 70 response-evaluable patients across all dose levels, 25 (36%) achieved a confirmed PR, 34 (49%) had SD, and 3 (4%) exhibited PD as their best response. Specifically, at the 100 mg BID dose, out of 36 response-evaluable patients, 14 (39%) had a confirmed PR, 17 (47%) had SD, and 1 (3%) had PD. For the phase I cohort treated with 100 mg BID (n = 13), longer follow-up data revealed a median duration of response and median progression-free survival exceeding 15 and 12 months, respectively. The safety profile was manageable, with most adverse events (AEs) being grade 1 and 2. Dose reductions and discontinuations due to AEs were infrequent, at 11% and 6%, respectively, for doses below 150 mg BID. Notably, no grade 3 or higher rash was observed at doses below 150 mg. These promising preliminary results led to Zipalertinib receiving breakthrough therapy designation from the US FDA.

### 3.5. Sunvozertinib

Sunvozertinib, an orally administered and potent TKI, specifically targets EGFR exon 20 insertion mutations, EGFR sensitizing mutations, T790M, and uncommon EGFR mutations, while demonstrating limited activity against wild-type EGFR. It exhibits selectivity for EGFR exon 20 insertion mutations, ranging from 1.4 to 9.6 times that of wild-type EGFR. Moreover, in patient-derived xenograft models, sunvozertinib’s oral administration resulted in significant anti-tumor activity, demonstrating dose-dependent efficacy [50].

Two phase I clinical trials, WU-KONG 1 and WU-KONG 2, are currently evaluating sunvozertinib for patients with metastatic NSCLC harboring EGFR or HER2 mutations. A pooled analysis assessed the drug’s safety, pharmacokinetics, and anti-tumor efficacy [37]. Among 56 patients with EGFR exon 20 insertion mutations, the confirmed ORR was 50% across all dose levels. The PFS rates at six months for the 100 mg, 200 mg, 300 mg, and 400 mg cohorts were 50%, 53.3%, 44.6%, and 44.4%, respectively, and had not yet reached the median. The treatment was well-tolerated, with all-grade diarrhea and rash occurring in 53.9% and 40.2% of patients, respectively. However, grade 3 or higher diarrhea occurred in only 4.9% of patients, and no patients experienced a rash of grade 3 or higher severity. Based on the safety and tolerability data from the dose-escalation cohorts, 400 mg was determined to be the maximum tolerated dose, with 200 mg to 400 mg selected for dose expansion [50,51]. In the WU-KONG 6 pivotal study, sunvozertinib demonstrated a confirmed ORR of 60.8% in Chinese patients with NSCLC with EGFR exon 20 insertion mutations [52].

## 4. Ongoing Clinical Trials (Table 3)

The NCT04077463 includes a specific focus on patients with NSCLC who have the EGFR exon 20 insertion mutation. This is a phase 1/1b study designed to assess the safety and pharmacokinetics of Lazertinib, a third-generation EGFR-TKI, both alone and in combination with Amivantamab, a bispecific antibody targeting EGFR and cMet. The trial includes multiple cohorts, one of which specifically targets patients with NSCLC characterized by the EGFR exon 20 insertion mutation. These patients are part of an expansion cohort, which aims to further characterize the safety, tolerability, and preliminary antitumor activity of Lazertinib and Amivantamab. Participants in this cohort are treated with the recommended phase 2 dose of Lazertinib and Amivantamab, administered every 7 days for the first 28-day cycle and then every 2 weeks thereafter, until disease progression or unacceptable toxicity.

**Table 3:** Ongoing clinical trials for patients with EGFR exon 20 insertion.

Agent	Trial Name	Study Population	Phase	NCT Number
Amivantamab	A Study of Lazertinib as Monotherapy or in Combination with Amivantamab in Participants with Advanced Non-Small-Cell Lung Cancer (CHRYSALIS-2)	Cohort B: previously treated with advanced or metastatic NSCLC with documented primary EGFR Exon 20ins activating mutation	I/Ib	NCT04077463
Amivantamab	A Study of Amivantamab in Participants with Advanced or Metastatic Solid Tumors Including Epidermal Growth Factor Receptor (EGFR)-Mutated Non-Small-Cell Lung Cancer (PALOMA-2)	Cohort 2: treatment-naive locally advanced or metastatic NSCLC harboring an EGFR exon20ins mutation	II	NCT05498428
Zipalertinib	A Study of Zipalertinib in Patients with Advanced Non-Small-Cell Lung Cancer with Epidermal Growth Factor Receptor (EGFR) Exon 20 Insertions or Other Uncommon Mutation (REZILIENT2)	Cohort A: patients harboring EGFR ex20ins mutations who have progressed on or after initial treatment with standard platinum-based chemotherapy and prior treatment with an ex20 agent for their advanced disease Cohort B: patients harboring EGFR ex20ins mutations who have not received prior treatment for advanced disease Cohort C: patients harboring EGFR ex20ins or other uncommon single and compound mutations and active brain metastases. Patients may or may not have had prior treatment for advanced disease	Iib	NCT05967689
Zipalertinib	A Study of Zipalertinib and Chemotherapy Compared with Chemotherapy Alone in Patients with Advanced Non-Small-Cell Lung Cancer with Epidermal Growth Factor Receptor (EGFR) Exon 20 Insertion (REZILIENT3)	Previously untreated, locally advanced or metastatic nonsquamous NSCLC harboring EGFR ex20ins mutations	III	NCT05973773

The NCT05498428 is focused on evaluating the efficacy and safety of Amivantamab in patients with advanced or metastatic solid tumors, including those with specific EGFR mutations such as the EGFR exon 20 insertion in NSCLC. The trial addresses the treatment of NSCLC patients who have the EGFR exon 20 insertion mutation and have experienced disease progression after platinum-based chemotherapy. Patients in this study receive Amivantamab administered subcutaneously, with the dosage and frequency determined by their body weight and treatment cycle specifics. The primary focus for this cohort is to assess the treatment’s safety profile and its efficacy in halting disease progression or managing the disease in a more tolerable manner.

The NCT05967689 is an open-label Phase 2b study assessing the safety and efficacy of the drug Zipalertinib in patients with advanced NSCLC that harbors specific EGFR mutations. This trial specifically targets patients with the EGFR exon 20 insertion mutation, as well as other less common EGFR mutations. The study includes multiple cohorts, with one cohort particularly focused on patients with active brain metastases. Participants in this cohort will receive Zipalertinib orally twice daily until disease progression or other criteria for withdrawal are met. Key outcomes being measured include the overall response rate, progression-free survival, overall survival, and the safety profile of the treatment, with additional specific measures for the impact on brain metastases in some cohorts.

The clinical trial identified by NCT05973773, known as REZILIENT3, is a phase 3 global, multicenter study evaluating the combination of zipalertinib and chemotherapy as a potential first-line treatment for adult patients with previously untreated, locally advanced or metastatic non-squamous NSCLC that have EGFR exon 20 insertion mutations. This trial aims to assess the efficacy and safety of zipalertinib in combination with chemotherapy compared to chemotherapy alone. The primary endpoints of the study are to evaluate the rate and severity of treatment-emergent adverse events and PFS. Secondary endpoints include the objective response rate, disease control rate, duration of response, overall survival, quality of life, pharmacokinetics, and EGFR mutation status.

## 5. Future Directions

In lung cancer with EGFR Exon 20 insertion mutations, brain metastases present a significant challenge, with studies like CHRYSALIS only including patients with previously treated asymptomatic brain metastases, showing varying rates of intracranial progression. Effective treatment of metastatic brain disease requires drugs that can overcome the blood–brain barrier (BBB), where transporters like P-glycoprotein significantly influence drug distribution. Promising results have been shown in preclinical studies with drugs like sunvozertinib, which exhibit good brain penetrance and encouraging antitumor activity, highlighting the need for further clinical trials to evaluate their efficacy in brain metastases. Future therapies must be designed to penetrate the CNS more effectively, and ongoing accumulation of clinical data is necessary to confirm the efficacy of current exon 20 insertion targeted therapies in treating intracranial lesions.

Furthermore, the landscape of resistance mutations in the context of EGFR exon 20 insertion targeted therapies is not fully understood. This gap underscores the need for enhanced detection strategies. The application of liquid biopsy techniques could play a pivotal role in the non-invasive detection of resistance mutations, providing a dynamic tool for monitoring disease progression and therapy resistance. After amivantamab treatment, molecular profiling of progressing patients revealed various genetic mutations. In the classical cohort, mutations such as MET amplification, MYC amplification, and loss of T790M were observed, while in the Ex20ins cohort, mutations like EGFR amplification and loss of AR mutation were noted, along with several new mutations [53]. Resistance to mobocertinib primarily stemmed from C797S and T790M mutations, which were more frequently observed in cells treated with lower drug concentrations [54]. Cells with the C797S mutation exhibited resistance to most EGFR TKIs, although erlotinib remained effective against some variants with C797S. Further research is needed to overcome these resistance mechanisms.

Finally, the integration of exon 20 insertion targeted therapies into first-line treatment represents a significant shift in the management of this mutation. Trials such as the PAPILLON study are exploring the efficacy of these therapies upfront. It is imperative to establish effective combination strategies and validate their therapeutic impacts in first-line settings to optimize outcomes for patients with this challenging mutation. These areas highlight the ongoing need for innovative research and robust clinical trials to improve the therapeutic landscape for patients with EGFR exon 20 insertion NSCLC. The use of both amivantamab and platinum-based cytotoxic chemotherapy in a first-line treatment setting reflects a comprehensive approach to manage aggressive and diverse tumor environments effectively. Combining it with platinum-based chemotherapy, which attacks cells more broadly by causing DNA damage, helps in targeting a wider range of tumor clones, thereby enhancing overall treatment efficacy. This strategy aims to reduce the likelihood of any single clone driving disease progression or developing resistance to treatment.

## Data Availability

Not applicable.
